# Cervicocephalic Spotty Calcium for the Prediction of Coronary Atherosclerosis in Patients With Acute Ischemic Stroke

**DOI:** 10.3389/fneur.2021.659156

**Published:** 2021-05-13

**Authors:** Chong Zheng, Shaozhen Yan, Fan Fu, Cheng Zhao, Daode Guo, Zhichao Wang, Jie Lu

**Affiliations:** ^1^Department of Radiology and Nuclear Medicine, Xuanwu Hospital, Capital Medical University, Beijing, China; ^2^Beijing Key Laboratory of Magnetic Resonance Imaging and Brain Informatics, Beijing, China

**Keywords:** acute ischemic cerebrovascular disease, atherosclerosis, calcium, coronary artery stenosis, computed tomography angiography

## Abstract

**Purpose:** To investigate the characteristics of cervicocephalic spotty calcium (SC) and coronary atherosclerosis in patients with acute ischemic stroke (AIS) and to assess the predictive value of SC for coronary atherosclerosis using combined coronary and cervicocephalic CTA.

**Materials and Methods:** Patients with AIS (*n* = 70) confirmed by brain MRI or CT and patients with asymptomatic carotid atherosclerosis (*n* = 58) confirmed by carotid ultrasonography were enrolled in our study. Subjects in both groups underwent combined coronary and cervicocephalic CTA. SC was used to evaluate cervicocephalic atherosclerosis. Coronary artery stenosis (CAS) ≥ 50% by segment and coronary artery calcium score (CACS) were used to evaluate coronary atherosclerosis. The SC frequency and the difference in coronary atherosclerosis between the two groups were compared, and the correlation between SC and coronary atherosclerosis was analyzed. Independent factors for CAS ≥ 50% were assessed via logistic regression analysis. Receiver operating characteristic curve analysis was performed to evaluate the added value of SC for predicting CAS ≥ 50%.

**Results:** Both SC and the CACS were significantly higher in the Stroke group than in the Control group (total SC count: 6.83 ± 4.34 vs. 2.98 ± 2.87, *P* < 0.05; CACS: 477.04 ± 798.01 vs. 136.31 ± 205.65, *P* < 0.05). There were significant differences in the presence of CAS ≥ 50% (61.4 vs. 27.6%, *P* < 0.001). SC and coronary atherosclerosis were significantly correlated for both the CACS and CAS ≥ 50% (*r* = 0.746 and 0.715, respectively; *P* < 0.001). SC was an independent predictor for CAS ≥ 50%.

**Conclusion:** SC correlates significantly with the CACS and could serve as an independent predictor of CAS ≥ 50% in patients with AIS, which suggests that combined cerebrovascular and cardiovascular assessments are of importance for such patients.

## Introduction

Atherosclerosis is a systemic disease that usually affects large and medium-sized arteries (including carotid and coronary arteries), can cause stroke and coronary artery disease (CAD) and is the main cause of death worldwide (1, 2). Approximately 52% of acute ischemic stroke (AIS) patients have asymptomatic CAD, two-thirds of AIS patients without a history of heart disease have more than 50% coronary artery stenosis (CAS), and 3% of patients have the risk of myocardial infarction (3, 4). Compared with the corresponding values for AIS patients without coronary atherosclerosis, the 2-year risk of CAD combined with vascular events is CAS ≥ 50%, but the 2-year risk of combined vascular events without cardiac symptoms is 4.36-fold higher (5). Therefore, screening and evaluating the CAD of AIS patients could help the clinical treatment and long-term prognosis of these patients and reduce the risk of CAD and AIS recurrence.

Cervicocephalic atherothrombotic occlusive vascular disease is a leading cause of AIS. Carotid plaque and increased carotid intima-media thickness are associated with the presence and severity of coronary calcification and disease (6, 7). However, the association between the carotid intima-media thickness progression assessed from two ultrasound scans and the cardiovascular risk in the general population remains unproven (8). Carotid plaque presence is a better predictor of CAD events than carotid intima-media thickness (9). On the other hand, vascular calcification is an important component of advanced atherosclerosis (10) and is a common sign that can be observed on computed tomography angiography (CTA). Intracranial artery calcifications correlate significantly with the coronary artery calcium score (CACS) and may serve as an independent predictor of asymptomatic CAD in patients with AIS (11). Fan and colleagues reported that cervicocerebral spotty calcium (SC) may provide a potential means for improving non-invasive risk stratification for AIS. Compared with subclinical atherosclerosis, AIS is associated with a high incidence of SC on cervicocephalic CTA (12). The findings from this study suggest the additional clinical value of the differential analysis of vascular calcium beyond a more general wholesale measure of the total amount of calcium. However, the potential association of SC with coronary atherosclerosis has not been well-investigated.

Previous evaluation methods for coronary and cervicocerebral arteries are different, and the evaluation indexes used are also inconsistent. With advancements in non-invasive imaging techniques, combined coronary and cervicocephalic CTA is feasible within a single acquisition, utilizing a single contrast media bolus (13). The purposes of this study to evaluate the characteristics of coronary and cervicocephalic atherosclerosis in patients with AIS and to investigate the relationship between SC and CACS, CAS using combined coronary and cervicocephalic CTA.

## Materials and Methods

### Patients

**Figure 1:**
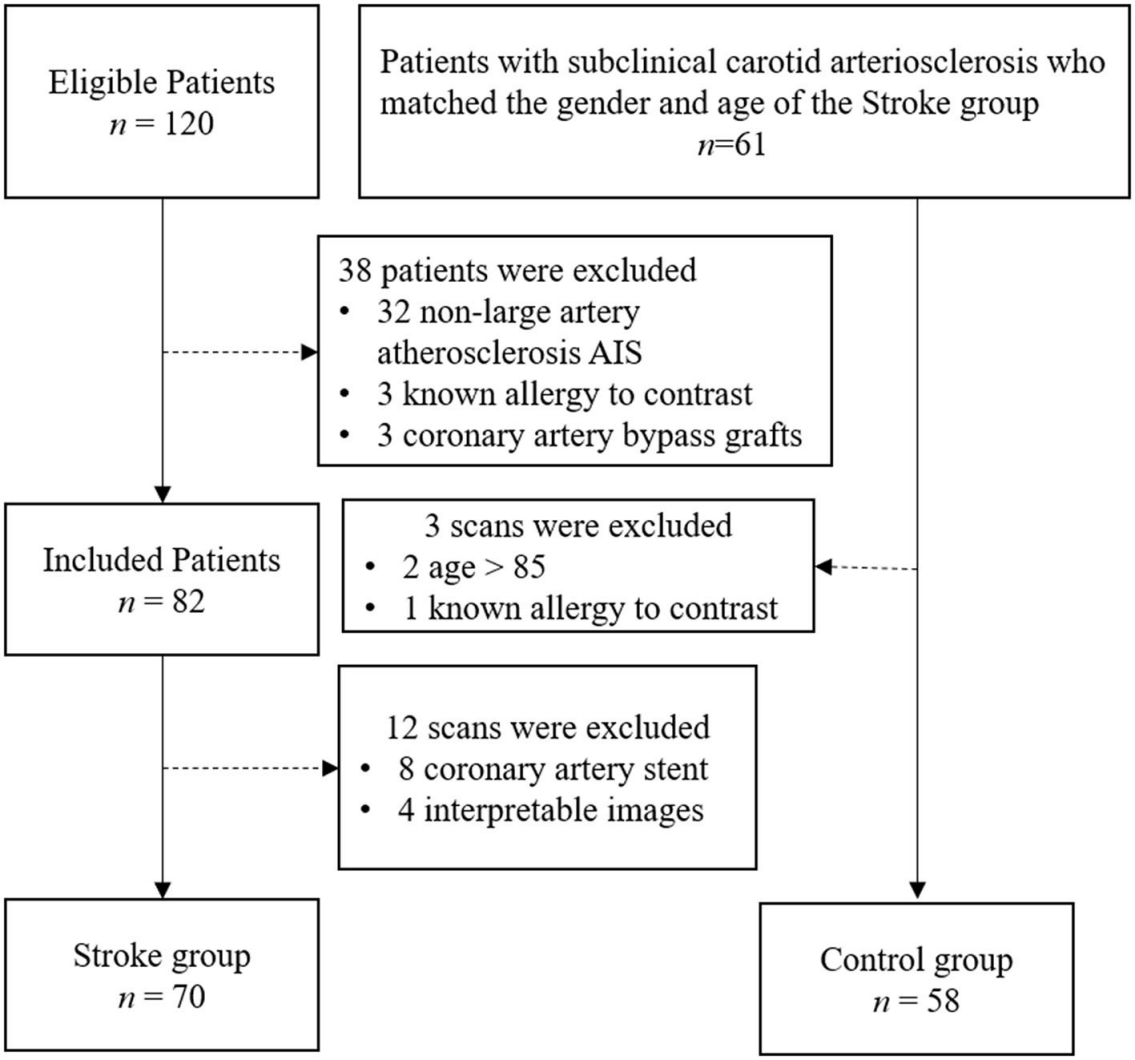
Flowchart of the patient enrolment.

The study was approved by the hospital ethics committee. Written informed consent was obtained from all patients or the appropriate family members. Patients who were admitted to our stroke unit from May 01, 2019, to January 30, 2020, were enrolled. Patients who met the following inclusion criteria were included in the Stroke group: (1) between 18 and 85 years old (2) diagnosed with AIS confirmed by the associated symptoms and imaging results from MRI or non-enhanced CT, according to American Heart Association or American College of Cardiology guidelines (14); (3) TOAST classification of AIS was Large-artery atherosclerosis. The exclusion criteria were as follows: (1) patients with a transient ischemic attack and lacunar ischemic stroke; (2) patients suspected cardioembolic stroke, evidence of cardioembolism (recent myocardial infarction <3 weeks, atrial fibrillation, mitral stenosis or prosthetic valve, dilated cardiomyopathy, sick sinus syndrome, acute bacterial endocarditis, patent foramen ovale, etc.), unexplained stroke; (3) non-atherosclerotic arterial stenosis, such as arterial dissection and vasculitis; (4) other pathogenesis such as hemathological diseases and tumor; (5) patients with coronary artery stents, angioplasty, or coronary artery bypass grafts; (6) patients who could not tolerate the CTA examination without interpretable images; and (7) patients who were allergic to iodine contrast material. Patients who met the following inclusion criteria were included in the Control group: (1) matched the sex and age of the Stroke group; (2) carotid arteriosclerosis disease diagnosed by carotid ultrasonography; (3) no ischemic stroke confirmed by MRI or CT; (4) no ischemic symptoms including transient global amnesia, acute confusion, syncope, bilateral weakness, paresthesia, etc. All ultrasound-confirmed carotid atherosclerosis patients were asked to undergo selective CTA to assess the overall carotid and coronary and cerebrovascular systems in more detail. All patients underwent combined coronary and cervicocephalic CTA within 7 days after the initial imaging examination. Finally, 128 patients were included in this study (Figure 1).

### Demographics and Clinical Characteristics

Demographic information, vascular risk factors (hypertension history, diabetes mellitus history, hypercholesterolemia history, smoking), and CAD history were recorded. Hypertension was defined by blood pressures ≥ 140/90 mmHg or current antihypertensive medication. Dyslipidemia was defined by a low-density lipoprotein cholesterol level ≥ 140 mg/dl, a high-density lipoprotein cholesterol level <40 mg/dl, a triglyceride level ≥ 150 mg/dl, or treatment with lipid-lowering medication. Diabetes was defined as a fasting blood glucose ≥ 126 mg/dl or HbAlc ≥ 6.5% or treatment with hypoglycemic medication. Patients were considered to have a smoking habit if they had actively smoked within the last 12 months before this hospital admission.

### CT Scan Protocols and Data Reconstruction

All CTAs were performed with a 256-MDCT scanner (Revolution CT, GE Healthcare, Milwaukee, USA). The maximal z-axis coverage range of the detector extended to 160 mm. The scanning range was from diaphragm to vertex for both groups. The scan sequence included localization images, CACS scans, coronary CTA scans, and cervicocerebral CTA scans completed after 2.2 s. In coronary CTA, all data were acquired using a prospective electrocardiogram-triggered axial scan during one tube rotation and within one R-R interval. The tube voltage was 100 kVp. The tube current was chosen automatically by a scanner, ranging from 350 to 650 mA. The preset noise index was 20 HU. The standard reconstruction type was applied with a hybrid iterative reconstruction algorithm (adaptive statistical iterative reconstruction-Veo, GE Healthcare) at a 50% blending percentage. In cervicocerebral CTA, the tube voltage was 100 kVp. The tube current was also chosen automatically, ranging from 300 to 600 mA. The preset noise index was 16 HU. The standard reconstruction type was applied with adaptive statistical iterative reconstruction-Veo at a 40% blending percentage.

The contrast agent application was controlled using the test-bolus technique. The contrast agent (Ioversol, 320 Iodine/ml; Bayer Schering Pharma, Germany) was injected intravenously through the antecubital vein by a power injector at a flow rate of 5 ml/s. A small dose of 15 ml contrast agent and 15 ml saline was injected to record the time density curve of the internal carotid artery and vertebral artery at the cervical 3–4 intervertebral space level. According to the peak time, the scan started 8 s later and 60 ml contrast agent were injected followed by 50 ml saline.

CT images were reconstructed with the image slice thickness and spacing of 0.625 mm. Reconstructed images were transferred to an external workstation (AW4.7, GE Healthcare) to evaluate all CTA datasets. CACS images were obtained with a 2.5-mm slice thickness from the carina to the bottom of the heart. The CACS from all calcified plaques in the coronary tree was calculated by an automated program according to the Agatston method (15). Curved planar reformatting, maximum intensity projection, multiplanar reformatting, and volume rendering images were used to evaluate the coronary and cervicocephalic arteries.

### Image Analysis

Cervicocephalic and coronary angiograms were reviewed by two certified radiologists blinded to the data. We selected 11 segments commonly affected by atherosclerosis, including both the carotid arterial system and the vertebrobasilar circulation. In the carotid arterial system, the left and right common carotid artery bifurcation, left and right siphon, and the first segment of the left and right middle cerebral artery were selected. In the vertebrobasilar circulation, the first and fourth segments of the left and right vertebral arteries (LVA1, RVA1, LVA4, and RVA4), as well as the basilar artery, were included in the analysis. In this study, calcium was defined as a region with ≥1 contiguous pixel with CT attenuation ≥ 130 Hounsfield units in each image slice (16). SC was defined based on a length of calcium (extent of the calcification parallel to the longitudinal direction of the vessel on the curved MPR image) below 3 mm and a maximum arc below 90° (12). According to the NASCET, the cervicocephalic arteries stenosis is divided into none (0), mild stenosis (1–49%), moderate stenosis (50–69%), severe stenosis (70–99%), and occlusion.

Coronary artery segments diameters > 2 mm were evaluated for the presence of atherosclerotic stenosis. Stenotic lesions were quantified for lumen diameter stenosis by visual estimation using a 5-point grading scale as follows: none (no luminal stenosis), minimal (1–24%), mild (25–49%), moderate (50–69%), severe (70–99%), or occluded, as per the guidelines of the Society of Cardiovascular Computed Tomography (17). CAS was evaluated by the severity and extent of stenosis. The presence of stenosis ≥ 50% in the coronary arteries and the grade of the most severe stenotic segment (0 for no stenosis; 1 for <50% stenosis; 2 for ≥50% and <70% stenosis; 3 for ≥70% stenosis; 4 for occlusion) were utilized to reflect the CAS severity. Patients with CAS ≥ 50% in only one or two coronary arteries and those with CAS in each of the three epicardial vessels were considered as having single-vessel disease/double-vessel disease and triple-vessel disease respectively, which yielded a measure of the CAS extent.

### Statistical Analysis

All statistical tests were performed using SPSS software (v22.0; IBM, Armonk, NY, United States). Quantitative variables are expressed as the mean ± standard deviation. Categorical variables were described by frequencies or percentages. Student's *t*-test was used to determine differences for normally distributed continuous variables, and the Mann-Whitney *U*-test was used for nonnormally distributed continuous variables. The chi-squared test was conducted for unordered categorical variables. Spearman's correlation coefficient was calculated to explore the association between variables. Multivariate linear regression analysis was employed to assess the value of SC to predict the coronary calcium score. Logistic regression was used to estimate the odds ratios of SC on coronary disease. A receiver operating characteristic (ROC) curve was plotted to determine the value of SC for discriminating coronary disease from normal conditions. A *P*-value < 0.05 was considered statistically significant.

## Results

### Patient Characteristics

The study population consisted of 128 patients (32 women and 96 men; mean age, 58.8 ± 10.7 years; age range, 32–85 years). Seventy patients (16 women and 54 men; mean age, 57.9 ± 11.2 years) were included in the stroke group, and the other 58 patients (16 women and 42 men; mean age, 59.8 ± 10.0 years) were included in the control group. Detailed clinical characteristics of the patient population enrolled in this study are included in Table 1. There was no significant difference between the 2 groups in age, BMI, smoking status, or other cardiovascular risk factors (*P* > 0.05).

**Table 1:** Clinical characteristics of the stroke group and control group.

**Characteristics**	**Stroke group**	**Control group**	***P***
	**(*n* = 70)**	**(*n* = 58)**	
Age (years)	57.9 ± 11.2	59.8 ± 10.0	0.30
Male (*n*, %)	54 (77.1)	42 (72.4)	0.54
BMI (kg/m^2^)	25.6 ± 3.6	25.2 ± 3.0	0.47
History of HTN (*n*, %)	47 (67.1)	38 (65.5)	0.85
History of HLP (*n*, %)	31 (44.3)	23 (40.0)	0.60
History of DM (*n*, %)	23 (32.9)	12 (20.7)	0.12
Smoking (*n*, %)	29 (41.4)	21 (36.2)	0.55
History of CAD (*n*, %)	9 (12.9)	6 (10.3)	0.66
History of ischemic stroke (*n*, %)	5 (7.1)	0 (0.0)	0.06

*BMI, body mass index; CAD, coronary artery disease; DM, diabetes mellitus; HLP, hyperlipidemia; HTN, hypertension*.

The Stroke group patients include 56/70 (80.0%) patients of intracranial atherosclerosis and 14/70 (20%) patients of extracranial atherosclerosis. And moderate cervicocephalic atherosclerotic stenosis was observed in 16/70 (22.9%) patients, severe stenosis was observed in 48/70 (68.5%) patients, and occlusion was observed in 6/70 (8.6%) patients.

### Stroke Group vs. the Control Group

**Figure 2:**
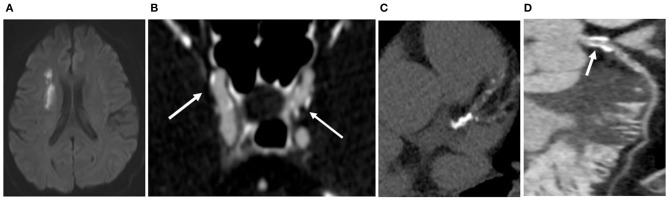
In the Stroke group, a 57-year-old man exhibited right basal ganglia region and lateral ventricle cerebral infarctions **(A)**. Cervicocerebral CTA showed multiple sites of SC (white arrow) at the siphonage of bilateral internal carotid artery **(B)** and the fourth segment of vertebral artery (a total of 10); multiple calcifications were detected on the coronary arteries **(C)**, and the total CACS was 689; CAS ≥ 50% was detected in the proximal LAD **(D)**, though the patient had never experienced chest symptoms.

**Figure 3:**
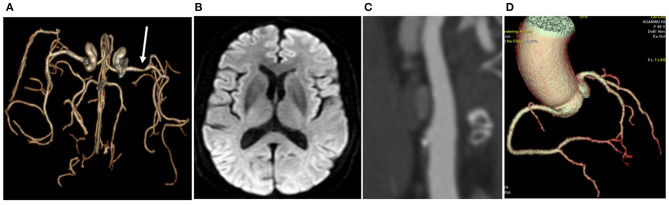
In the Control group, a 69-year-old woman had left middle cerebral artery stenosis **(A)**. No infarction was found by MR **(B)**; only one area of SC (white arrow) was detected at the bifurcation of the right common carotid artery **(C)**; no calcification was observed in the coronary artery, the calcification score was 0, and no stenosis was detected in the coronary artery **(D)**.

The numbers of total SCs, common carotid artery bifurcation SCs, internal carotid artery siphon SCs, VA1 SCs and VA4 SCs in the Stroke group were higher than those in the Control group (all *P* < 0.05) (Table 2, Figures 2, 3). There was no significant difference in SC in the remaining vascular segments between the two groups. The segments with the most SC involvement in the Stroke group were the siphons of the bilateral internal carotid artery (3.39 ± 2.30), followed by the bifocals of the common carotid artery (2.34 ± 2.18). The segments with the most SC involvement in the Control group were the siphons of the bilateral internal carotid artery (1.41 ± 1.70). No spotty calcification was observed at middle cerebral arteries Only 1 patient in the Stroke group had spotty calcification in the basilar artery.

**Table 2:** Comparison of SC and the CACS between the stroke group and the control group.

	**Stroke group**	**Control group**	***P***
	**(*n* = 70)**	**(*n* = 58)**	
Total spotty calcium	6.83 ± 4.34	2.98 ± 2.87	< 0.001^*^
Carotid bifurcation, bilateral	2.34 ± 2.18	1.41 ± 1.70	0.011^*^
Siphon, bilateral	3.39 ± 2.30	1.16 ± 1.06	< 0.001^*^
VA1, bilateral	0.33 ± 0.61	0.03 ± 0.18	< 0.001^*^
VA4, bilateral	0.76 ± 1.29	0.43 ± 1.06	0.030^*^
Total CACS	477.04 ± 798.01	136.31 ± 205.65	0.003^*^
LM and LAD of CACS	242.84 ± 374.51	87.45 ± 136.22	0.011^*^
LCX of CACS	65.66 ± 138.38	20.04 ± 43.75	0.015^*^
RCA of CACS	168.85 ± 376.17	28.82 ± 65.81	< 0.001^*^

* *P < 0.05 was considered statistically significant*.

*CACS, coronary artery calcium score; LAD, left anterior descending; LCX, left circumflex; LM, left main trunk; RCA, right coronary artery; VA, vertebral artery*.

The CACS in the Stroke group was higher than that in the Control group (477.04 ± 798.01 vs. 136.31 ± 205.65, *P* < 0.05) (Figures 2, 3). Each coronary artery was analyzed separately, and a significantly higher CACS was observed in the Stroke group at each of the three evaluated vascular locations (Table 2).

Compared with the Control group, the group with AIS was more likely to have CAS ≥ 50% (61.4 vs. 27.6%, *P* < 0.001), and the grade of the most severe stenotic segment tended to be larger (median 2 vs. 1, *P* < 0.001). Regarding the CAS extent characteristics, there were significant differences in single-vessel disease, double-vessel disease and triple-vessel disease between the two groups (*P* < 0.001) (Table 3).

**Table 3:** Comparisons of CAS characteristics between the stroke group and the control group.

**Characteristics**	**Stroke group**	**Control group**	***P***
	**(*n* = 70)**	**(*n* = 58)**	
Presence of CAS ≥ 50% (*n*, %)	43 (61.4)	16 (27.6)	< 0.001^*^
CAS extent (*n*, %)			< 0.001^*^
No CAS ≥ 50%	27 (38.6)	42 (72.4)	
Single-vessel disease	16 (22.9)	11 (19.0)	
Double-vessel disease	19 (27.1)	4 (6.9)	
Triple-vessel disease	8 (11.4)	1 (1.7)	
Grade of the most severe stenotic segment [M (Q25, Q75)]	2 (1, 3)	1 (0, 2)	< 0.001^*^

* *P < 0.05 was considered statistically significant*.

*CAS, coronary artery stenosis*.

### Correlation of Atherosclerosis Between Coronary and Cervicocephalic Arteries

There was a significant correlation between SC and coronary atherosclerosis. The Spearman correlation coefficient between SC and the CACS in all patients was 0.746 (*P* < 0.001), while that between SC and CAS ≥ 50% was 0.715 (*P* < 0.001). In the Stroke group, the Spearman correlation coefficient between SC and CACS was 0.747 (*P* < 0.001), while that between SC and CAS ≥ 50% was 0.690 (*P* < 0.001). In the Control group, the Spearman correlation coefficient between SC and the CACS was 0.655 (*P* < 0.001), while that between SC and CAS ≥ 50% was 0.614 (*P* < 0.001).

According to the linear regression model with adjusted baseline parameters including age, hypertension, hyperlipidemia, diabetes mellitus, body mass index and smoking, total SC was a predictor of the CACS (*P* < 0.001). According to the logistic regression model with adjusted baseline parameters including age, hypertension, hyperlipidemia, diabetes mellitus, body mass index, and smoking, total SC was a significant predictor of CAS ≥ 50% (Table 4).

**Table 4:** Logistic analysis for predicting CAS ≥ 50%.

	**β**	**SE**	**OR (95%CI)**	***P***
Total SC of Stroke group	0.852	0.243	2.344 (1.455–3.778)	< 0.001^*^
Total SC of Control group	1.160	0.428	3.189 (1.378–7.380)	0.007^*^
Total SC of all patients	0.808	0.164	2.243 (1.625–3.096)	< 0.001^*^

* *P < 0.05 was considered statistically significant*.

*β, Logistic regression coefficient; CI, confidence interval; OR, odds ratio; SC, spotty calcium; SE, standard error*.

**Figure 4:**
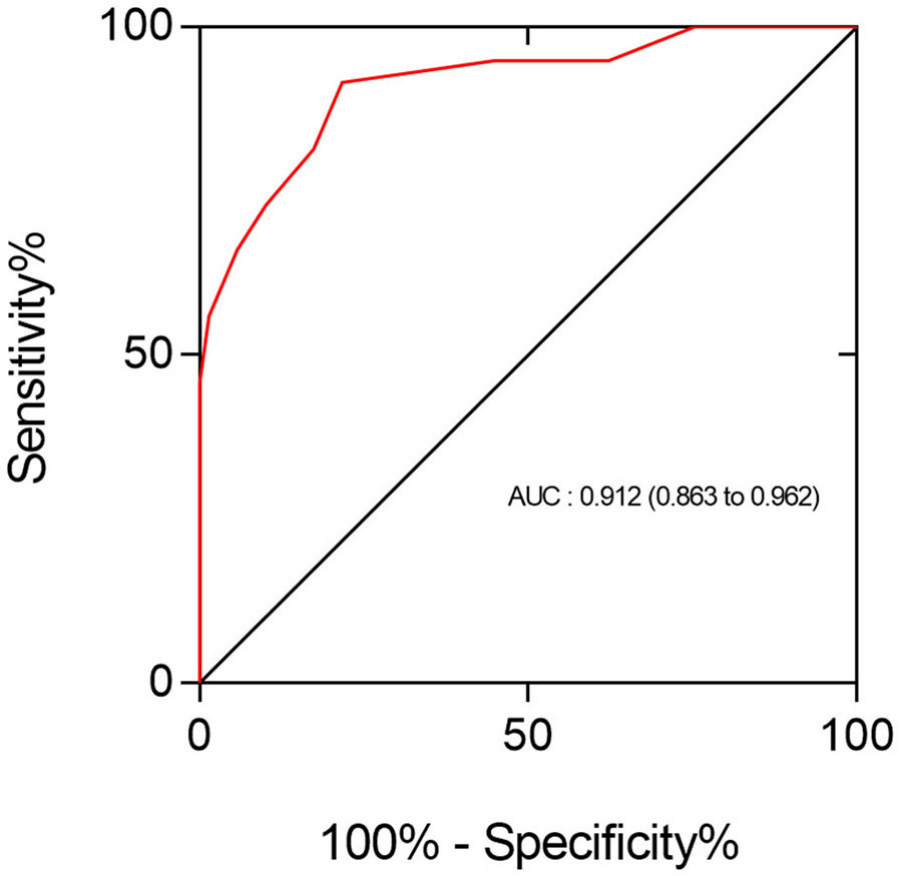
The ROC analysis showed that the performance of total SC in identifying the presence of CAS ≥ 50% in all patients was good (AUC = 0.912; 95% CI: 0.862–0.962). The optimal cutoff point of SC for discriminating these two types of coronary conditions was 3.5, yielding a sensitivity of 92% and specificity of 78%.

Figure 4 below illustrates the recognition performance of total SC for the presence of CAS ≥ 50% in all patients. In the Stroke group, ROC analysis determined that a total number of 5.5 SC was the optimal cutoff threshold for diagnosing the presence of CAS ≥ 50%, corresponding to sensitivity and specificity values of 0.860 and 0.815, respectively. In the Control group, the optimal cutoff threshold with total SC to determine the presence of CAS ≥ 50% was 3.5, corresponding to a sensitivity of 0.875 and specificity of 0.857. The diagnostic value of SC in the Stroke group was better than that in the Control group, with the former showing an AUC of 0.908 (95% CI: 0.840–0.976) vs. an AUC of 0.891 (95% CI: 0.800–0.983) for the latter.

## Discussion

This study was designed to investigate the correlation between coronary and cervicocephalic atherosclerosis through SC, the CACS and CAS in patients with AIS. The SC value of the cervicocephalic CTA determined whether the patient needed extra coronary CTA.

Atherosclerosis is a systemic disease. There is strong evidence to show that cervicocephalic artery atherosclerosis is associated with coronary atherosclerosis (9). Previous studies on the correlation between carotid artery and coronary atherosclerosis mostly used carotid Doppler ultrasonography and the CACS. Neman et al. found that the CACS had significant positive correlations with total and segmental carotid intimal medial thickness in black and white middle-aged men (18). Lee et al. observed that the CACS was independently associated with overt carotid stenosis and carotid plaque surface irregularity regardless of ethnicity (19). Studies have also assessed the correlation between the cervicocephalic artery and coronary artery calcification obtained by CT. In a study of 600 patients, Odink et al. found a moderate correlation between cervicocephalic artery and coronary artery calcification (*r* = 0.55, *P* < 0.001) (20).

Vascular calcification plays an important role in the progression of atherosclerosis, occurring in up to 90% of atherosclerotic lesions (21). Although the pathophysiological mechanism of vascular calcification is incompletely understood, there is a large amount of evidence supporting the association between cervical cerebrovascular calcification and neurovascular events, including ischemic stroke and lacunar infarction (22). Chen et al. reported in a cross-sectional study that the incidence of intracranial arterial calcification was higher in patients with ischemic stroke in an Asian cohort and that the presence of intracranial arterial calcification is an independent risk factor for ischemic stroke (23). Several studies have demonstrated that the amount of cavernous calcification between acute stroke and age-matched control groups is not different, which also shows that the clinical significance of cervical cerebrovascular calcification is still controversial (24). Previous studies investigating the clinical significance of cervicocerebral vascular calcification have mainly focused on the incidence and total amount of calcium without drawing clear distinctions between different types of arterial calcium. Therefore, the clinical significance of calcium may be controversial. Furthermore, SC is one of the characteristics of vulnerable coronary artery plaques, suggesting the instability of the plaques, but studies on SC in the cervicocephalic arteries are rare. In this study, SC was used as an indicator to evaluate the plaque burden of cervicocephalic atherosclerosis. The total SC of patients in the Stroke group was significantly higher than that in the Control group (6.83 ± 4.34 vs. 2.98 ± 2.87, *P* < 0.001). This finding is in accordance with the study by Fan et al. (12). A study of 445 patients with lacunar cerebral infarction found that calcification in the internal carotid artery siphon was positively correlated with the occurrence of lacunar infarct and that the degree of calcification in the internal carotid artery siphon could be used as a predictive risk factor (25). A 6-year follow-up study of 14,055 people in Europe confirmed that intracranial calcification of the internal carotid artery was a major risk factor and that intracranial carotid artery calcification contributed to 75% of all strokes; for aortic arch and extracranial carotid artery calcification, this incidence was only 45 and 25%, respectively (26). Compared to that studies, the present study has following strengths: it was found that the total amount of SC in patients in the Stroke group was significantly higher than that in the Control group, and the SC values of all vascular segments in the Stroke group were also higher. Moreover, there was a significant difference between the two groups in the internal carotid artery siphon SC, suggesting that calcification in the internal carotid artery siphon was helpful in predicting the risk of stroke.

CAD is an important factor influencing the prognosis of patients with non-fatal ischemic stroke. Yoo et al. showed that stroke patients with asymptomatic CAD on CT have greater risks of future major vascular events than those without (4). In addition, their study demonstrated that the risk of major vascular events increased as the extent of CAS increased in patients with AIS. CACS can independently predict CAD and provide better prognostic value. (27, 28) In this study, the patient's coronary atherosclerosis were evaluated based on CACS and CAS. The CACS in the Stroke group was higher than that in the Control group, and the CACS in each coronary artery branch was also higher than that in the Control group. In addition, the severity and extent of CAS were also evaluated. The presence of CAS ≥ 50%, degree of stenosis, and extent of CAS ≥ 50% in the Stroke group were higher than those in the Control group (*P* < 0.05). Therefore, for patients with AIS, the risk of combined CAD should not be ignored. CAD is an independent risk factor for poor prognosis in patients with AIS. Coronary artery evaluation of patients with AIS can screen out high-risk patients prone to cardiovascular events at an early stage, which is of great significance for improving the prognosis of patients.

In this study, combined CTA was applied to obtain the cervicocephalic artery and coronary artery simultaneously in one examination. Our research found that the SC was significantly correlated with the CACS (*r* = 0.747, *P* < 0.001). Furthermore, our study revealed more comprehensive findings. We showed a strong association between the total SC and CAS ≥ 50% (*r* = 0.690, *P* < 0.001). The strength of our study is that it provided the best cutoff value for SC to discriminate coronary involvement from normal coronary conditions. In this regard, the cutoff was 5.5 for SC, yielding acceptable sensitivity and specificity for predicting coronary disease. Therefore, through the analysis of the SC value of AIS patients, we can infer the patient's need for coronary CTA and reduce the radiation dose received by the patient and the amount of iodine contrast agent. Moreover, SC is likely to present as calcified nodules in lipid-rich plaques that have extensive positive remodeling, while thick, large pieces of calcium are more likely to present as fibrocalcific plaques with thick fibrous caps overlying extensive calcium in the intima (12). It is hereby certified that a specific analysis of the calcification morphology may be superior to the evaluation of total calcium by the calcification score alone. The results of this study suggest that SC provides additional information for predicting the coronary atherosclerosis burden, and there is a certain correlation between cervicocephalic and coronary atherosclerosis.

There are several limitations in this study. First, this was a single-center study consisting of a limited number of patients with AIS or subclinical carotid arteriosclerosis. Second, AIS is a heterogeneous disease, there are five types of AIS according to TOAST (29). Hence, the prediction results may be different depending on the etiologies of stroke. Third, although the AUC is considered to indicate the probability of correct decision making and can reflect the predictive value of a diagnostic test to some extent, it cannot completely replace the assessment of predictive ability. Therefore, the current results require a future large randomized trials should be conducted to evaluate the prognostic value of SC.

## Conclusion

In summary, the burden of atherosclerosis in the cervicocephalic arteries and coronary atherosclerosis in patients with AIS was higher than that in patients with subclinical atherosclerosis. SC correlated significantly with the CACS and was an independent predictor for the presence of CAS ≥ 50% quantified by using CTA. Combined coronary and cervicocephalic CTA can effectively evaluate the degree and relationship of atherosclerosis between cervicocephalic arteries and coronary arteries, providing important value for clinical guidance and treatment.

## Data Availability Statement

The raw data supporting the conclusions of this article will be made available by the authors, without undue reservation.

## Ethics Statement

The studies involving human participants were reviewed and approved by Xuanwu Hospital ethics committee. The patients/participants provided their written informed consent to participate in this study. Written informed consent was obtained from the individual(s) for the publication of any potentially identifiable images or data included in this article.

## Author Contributions

Study was designed by JL, CZha, and CZhe. Material preparation, data collection, and analysis were performed by CZhe, CZha, DG, and ZW. The first draft of the manuscript was written by CZhe. SY, FF, and JL provided critical review. All authors contributed to the study conception and design and read and approved the final manuscript.

## Conflict of Interest

The authors declare that the research was conducted in the absence of any commercial or financial relationships that could be construed as a potential conflict of interest.
